# Tumor cell identification and classification in esophageal adenocarcinoma specimens by hyperspectral imaging

**DOI:** 10.1038/s41598-022-07524-6

**Published:** 2022-03-16

**Authors:** Marianne Maktabi, Yannis Wichmann, Hannes Köhler, Henning Ahle, Dietmar Lorenz, Michael Bange, Susanne Braun, Ines Gockel, Claire Chalopin, René Thieme

**Affiliations:** 1 Innovation Center Computer Assisted Surgery (ICCAS), Leipzig University, Leipzig, Germany; 2 Department of Visceral, Transplant, Thoracic and Vascular Surgery, University Hospital of Leipzig, Leipzig, Germany; 3 Department of General and Visceral Surgery, Sana Clinic Offenbach GmbH, Offenbach, Germany; 4 Department of General, Visceral and Thoracic Surgery, Municipal Hospital of Darmstadt GmbH, Darmstadt, Germany; 5 Institute of Pathology, Sana Clinic Offenbach GmbH, Offenbach, Germany

**Keywords:** Gastrointestinal cancer, Oesophageal cancer

## Abstract

Esophageal cancer is the sixth leading cause of cancer-related death worldwide. Histopathological confirmation is a key step in tumor diagnosis. Therefore, simplification in decision-making by discrimination between malignant and non-malignant cells of histological specimens can be provided by combination of new imaging technology and artificial intelligence (AI). In this work, hyperspectral imaging (HSI) data from 95 patients were used to classify three different histopathological features (squamous epithelium cells, esophageal adenocarcinoma (EAC) cells, and tumor stroma cells), based on a multi-layer perceptron with two hidden layers. We achieved an accuracy of 78% for EAC and stroma cells, and 80% for squamous epithelium. HSI combined with machine learning algorithms is a promising and innovative technique, which allows image acquisition beyond Red–Green–Blue (RGB) images. Further method validation and standardization will be necessary, before automated tumor cell identification algorithms can be used in daily clinical practice.

## Introduction

Worldwide, esophageal cancer is the sixth leading cause of cancer-related death^1^. Despite improved treatment algorithms for esophageal adenocarcinoma (EAC), patients have a moderate histological response after neoadjuvant chemotherapy, with only 16% showing a complete tumor regression^2^. Therefore, there is an urgent need to improve the overall survival (OS). However, this is highly dependent on patients’ tumor, lymph node and metastasis (TNM)-category, with the best OS in T1 patients^3^. Tumor diagnosis is driven by the histopathological investigation of tumor specimens. The discrimination between malignant and non-malignant cells in histological specimens is mandatory for cancer diagnosis. Artificial intelligence (AI) might be an efficient tool to minimize the labor time and costs for pathological diagnosis and to straighten decision-making. However, it will not replace an independent supervision of an experienced pathologist. Thus, histopathologic evaluation will still remain the “gold standard” of diagnosis.

Hyperspectral imaging (HSI), a novel technology combining imaging with spectroscopy, allows the investigation of a spectrum from the visual to near-infrared light (500–1000 nm). The advantage of HSI over conventional imaging is a three-dimensional dataset of spatial and spectral information, called hypercube image data^4^. Machine learning algorithms are powerful tools for cancer cell identification and classification using hyperspectral data^5^. Recently, these algorithms combined with HSI technology have been applied for head and neck cancer^6^, gastric cancer^7^, breast cancer^8^, and prostate cancer^9^. All of these studies have implemented very few well-defined patients only, which do not reflect the daily pathological routine. However, HSI have been shown a high sensitivity, specificity, and accuracy of 71%, 98%, and 85%, respectively^7^. HSI data with their complex and comprehensive hypercube structure are a predestined source for machine learning algorithms. Several of these have been shown to support the identification and classification of cancer cells in HSI data^7,9–11^. Algorithms to distinguish between cancerous and non-cancerous tissue in pathological slides have been used for gastric and pancreatic tumors already^12,13^, brain tumors^14^, oral cancer^15^, thyroid carcinomas^16,17^, breast cancer^18^ and liver tumors^19^.

In this work, we classified pixel-wise three different histopathological features (cells from squamous epithelium, EAC, and tumor stroma) based on machine learning methods. A multi-layer perceptron (MLP) with two hidden layers was used to separate the three classes in HSI images of histopathological specimens from 95 patients, who had undergone oncologic esophagectomy for EAC.

## Results

### Image generation, feature reduction, and processing

**Figure 1:**
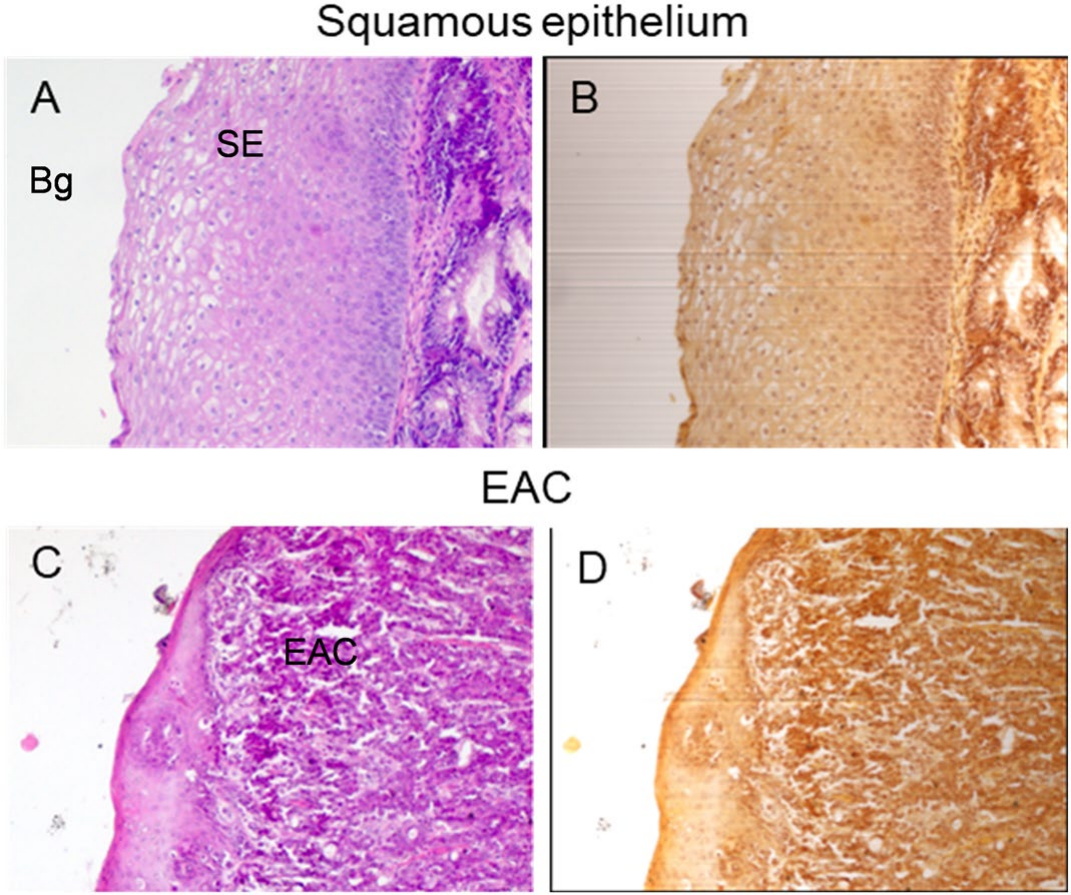
Hematoxylin and eosin (HE) stained specimens. Specimens were stained for HSI imaging standardizedly. Areas with squamous epithelium (**A**) or EAC cells (**C**) were selected and recorded by the HSI camera. The corresponding RGB images reconstructed from the HSI data are shown in (**B**) and (**D**) (*Bg* Background, *SE* Squamous epithelium and *EAC* Esophageal adenocarcinoma cells).

EAC specimens with tumor cell rich areas, which were identified with conventional light microscopy, were selected to be analyzed by HSI. A pathologist supervised selection of tumor cell bearing specimens. Afterwards, the HSI camera (500–1000 nm) recorded the identified region of interest. Thereby, a differentiation of background (Bg), squamous epithelium (SE), EAC and tumor stroma cells can be done based on the synthesized RGB image from (Fig. 1). Conventional RGB images (Fig. 1A/C) were recorded to demonstrate the similarity of the cellular structure in the synthesized RGB images from HSI.

**Figure 2:**
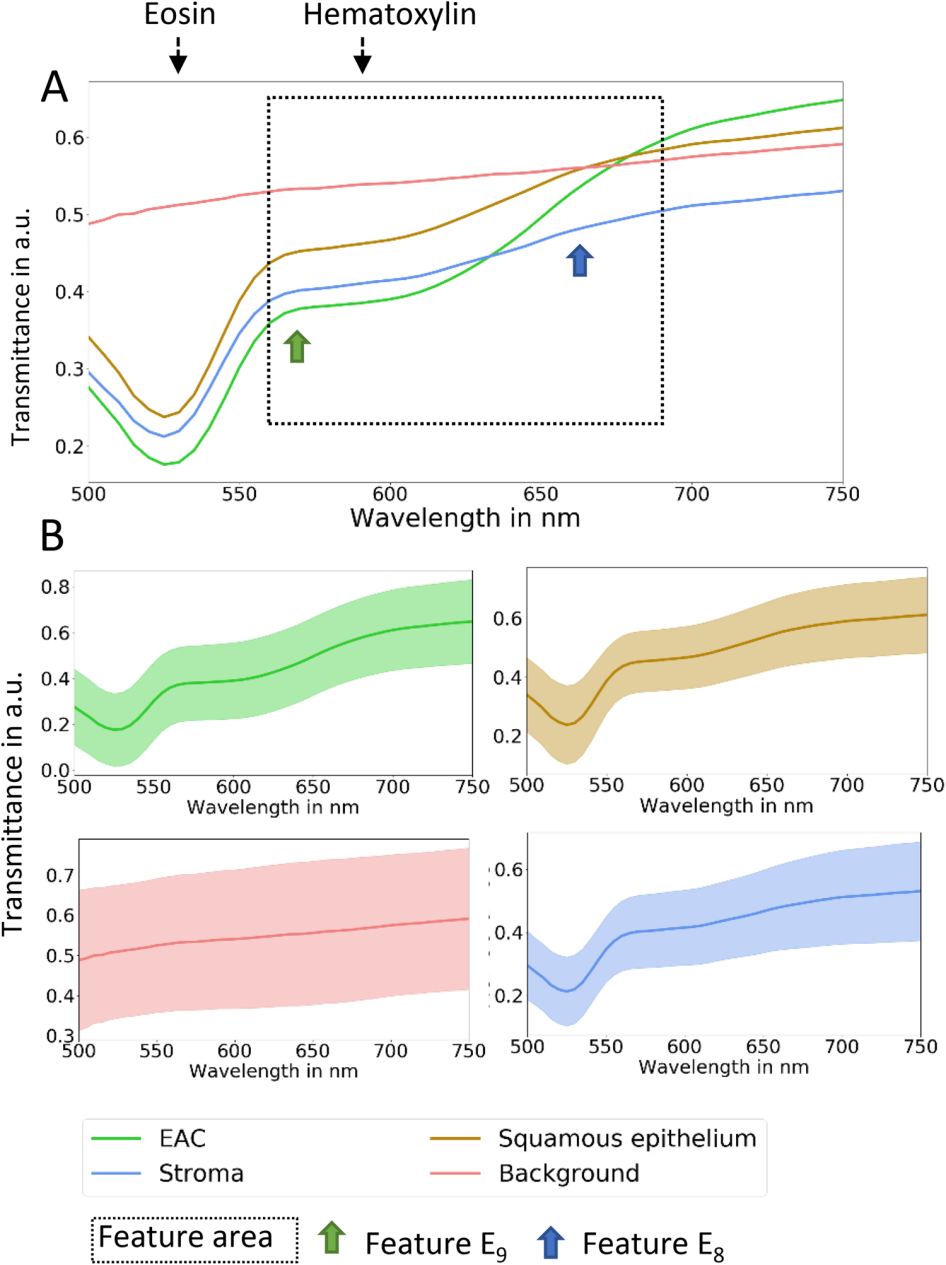
The mean transmittance of the spectra values of the four different classes. (**A**) The black-dotted arrows show the wavelengths of the transmittance peaks of hematoxylin and eosin. The dotted frame shows the area of the features, which were used to classify the samples. The green and blue arrows depict the feature E7 and E8, respectively. The transmittance of the background reveal a high difference to the other classes. (**B**) Mean and standard deviation of the standard normal variate (SNV) of transmittance of squamous epithelium, EAC, tumor stroma cells, and background are shown. All classes have a high standard deviation over the whole wavelength range. (a.u. - aberrante units)

**Figure 3:**
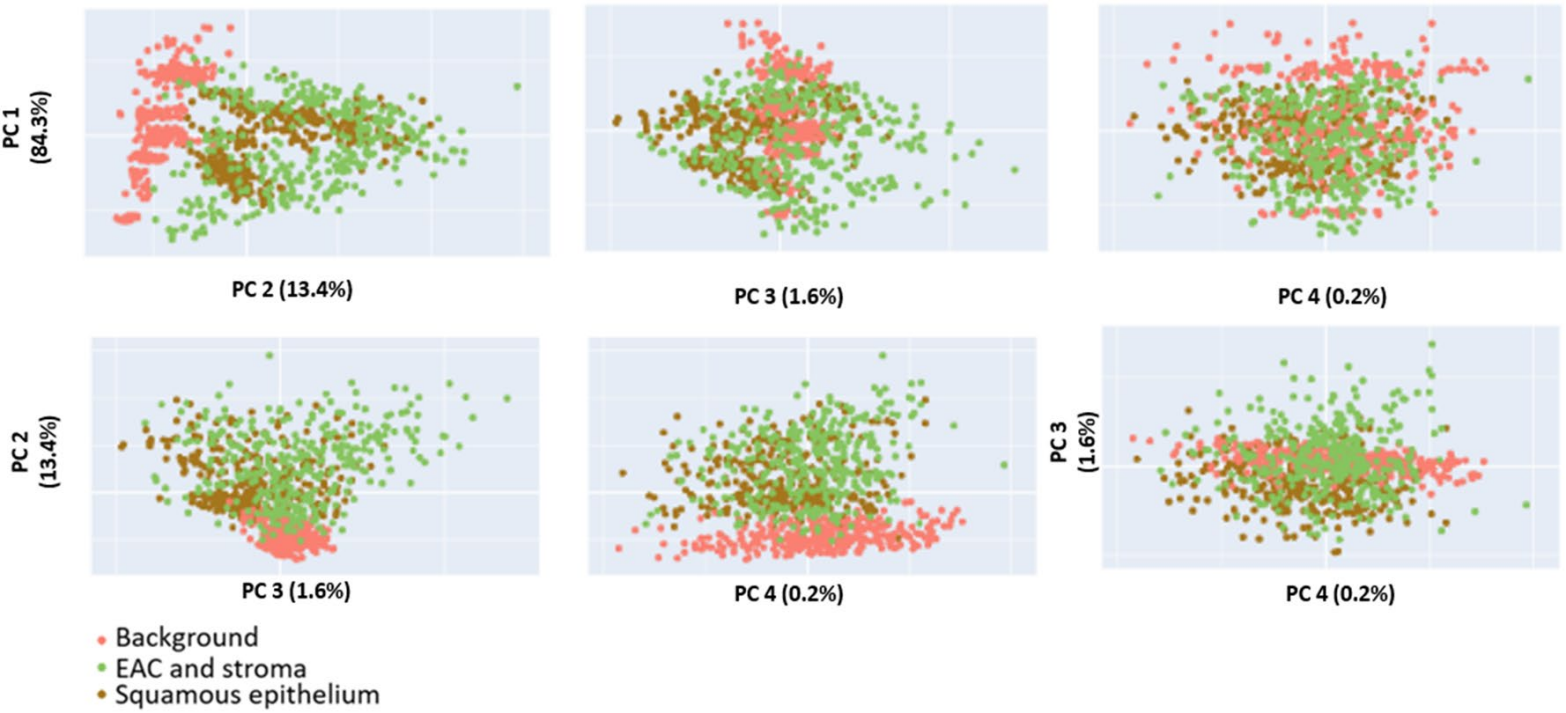
Principal component analysis (PCA) of the four classes were performed with MATLAB R2018b to analyze the class-variance. To show the clusters of the classes, we used only 1000 spectral data of each class. The data were randomly selected.

The spectra of the respective regions of interest were exported as annotated data for further pixel-wise classification process. The regions of interest were allocated to squamous epithelium, tumor stroma, cancer cells and background, and were investigated separately. After the annotation process, we obtained data for classification in total, 507,419 spectra. 55,711 spectra of squamous epithelium cells, 412,964 spectra of EAC cells, 32,318 spectra of tumor stroma cells, and 6426 spectra of background, were used for the classification. A differentiation against the four classes, squamous epithelium cells against tumor stroma cells, EAC cells, and background is crucial. Principal component analysis, calculation, and visualization of the mean and standard deviation were done, to analyze the differences of the spectra of the four classes (Figs. 2 and 3).

The spectra showed high standard deviation values and similar mean values, whereby the peaks of hematoxylin and eosin were visible for squamous epithelium, EAC, and stroma cells. Only the background had a linear spectrum, because the HE staining showed no influence on the spectra in the range from 500 to 750 nm (Fig. 2). The standard deviation was high due to the high patient-inter and -intra variability of chemical components in tissue, as well as the SNR of the HSI system.

Principal component analysis (PCA) of the four classes was performed with MATLAB R2018b to analyze the class-variance. To show the clusters of the classes, we used only 1,000 spectral data of each class. Data were randomly selected. The features EAC and stroma were merged together, due to their similarity in cell structure. In further preprocessing and classification steps, this merging of the two featues was proceeded. The interference of the first fourth principal components of the structures squamous epithelium, EAC, tumor stroma, and background was very high (Fig. 3). The PCA results (first component 84%, second component 13%, and third component 2%) demonstrated that the different classes were not separable by using the PCA components. Therefore, this feature selection method was not suitable to distinguish between the structures squamous epithelium, EAC, tumor stroma and background.

**Figure 4:**
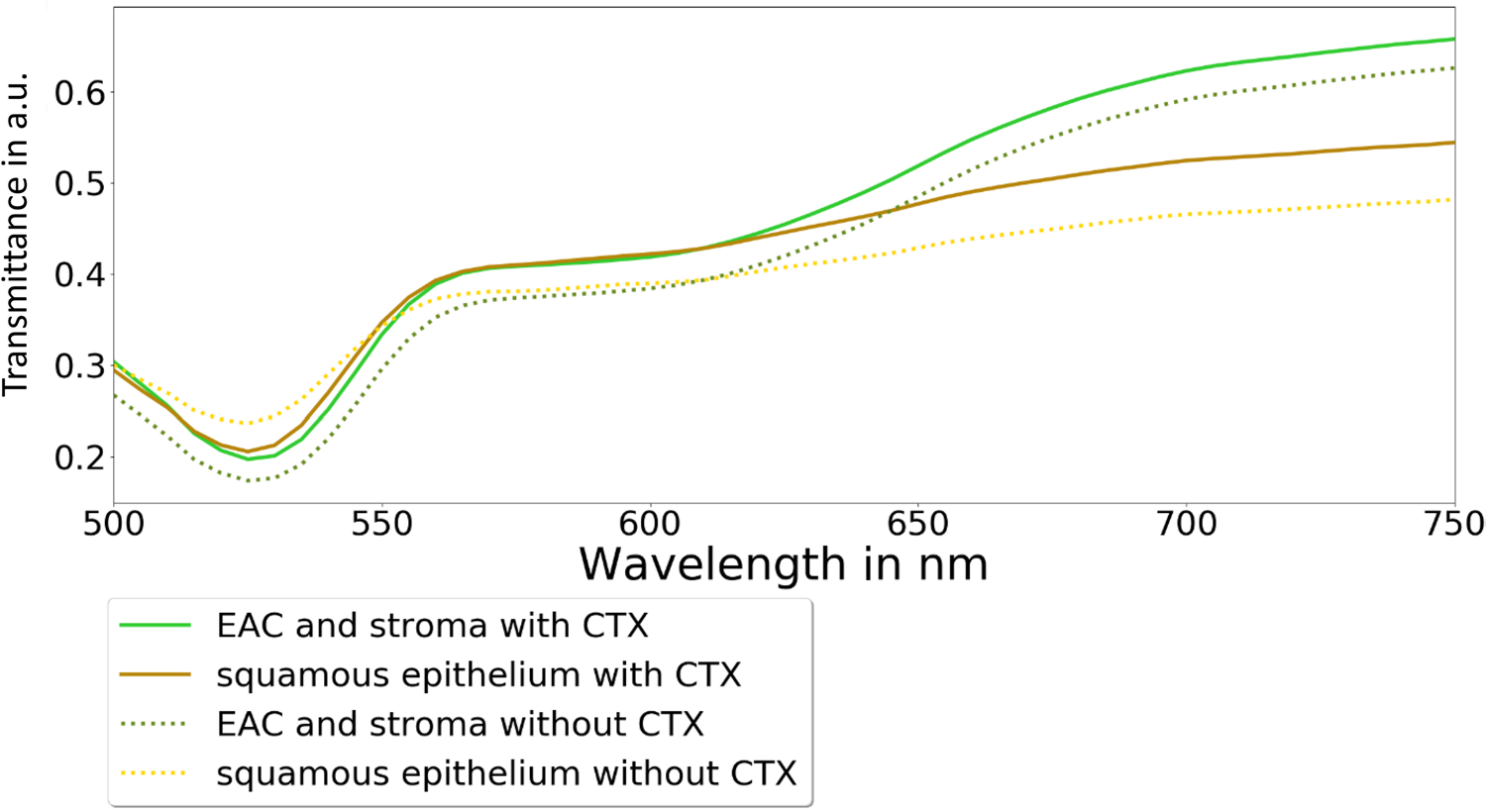
The mean transmittance of the spectra values of patients with and without neoadjuvant chemotherapy (CTx) is shown. The EAC and stroma without CTx versus squamous epithelium without CTx showed a high difference in the area from 500 to 570 nm and from 650 to 750 nm. The EAC and stroma with CTx versus squamous epithelium with CTx showed a high difference only in the area from 650 to 750 nm.

Furthermore, the spectra of patients with and without neoadjuvant chemotherapy were analyzed to determine the differences of cellular characteristics after chemotherapy by HSI. The spectra differed especially in the area of the eosin peak at the absorption maximum of 525 nm (Fig. 4).

### Classification

In this work, a multi-layer perceptron (MLP) with four layers and two hidden layers were used. Eight features or three RGB channels, which are described in the section “Feature extraction”, were used for the pixel-wise classification. A preliminary test using a leave-one-patient-out cross validation (LOPOCV) with 20 patients was performed by using a hyperaparmeter optimization algorithm based on Bayesian Optimization. The support vector machine (SVM), logistic regression (LR) and MLP showed similar results, but with several training-test sets grouped by patients (totally 10 training-test sets, where 70% was used for training and 30% was used for testing), SVM and LR showed a high variance of the best suitable hyperparameters. Because the MLP showed the lowest variance of the best-fitted hyperparameters, MLP was chosen as algorithm for the whole data set. Four different methods were used in a LOPOCV with:

- All patients (*n* = 95) (evaluating with a training set (*n* = 94) and a test set (*n* = 1) for each patient)
- patients without neoadjuvant therapy (*n* = 46) (evaluated with a training set (*n* = 45) and a test set (*n* = 1) for each patient)
- patients with neoadjuvant therapy (*n* = 49) (evaluated with a training set (*n* = 48) and a test set (*n* = 1) for each patient) and
- all patients (*n* = 95) using the RGB channels only; the synthetic RGB image was calculated based on the RGB channels (evaluated with a training set (*n* = 94) and a test set (*n* = 1) for each patient).

The LOPOCV was done to simulate a realistic practical use case. The tested model was not trained on the same subject.

The classification performance for the combined class of EAC and tumor stroma cells, as well as for the class background was high, with F1-score, sensitivity, and specificity values > 77% (Table 1). The squamous epithelium was, thus, more difficult to be classified correctly. However, the F1-score was 53%, the sensitivity was 54%, and the specificity was 81%, when the classification was performed on the HSI data of the 95 patients. To investigate the impact of potential changes in cell morphology due to neoadjuvant treatment, the cohort was subgrouped into specimens with and without neoadjuvant treatment. Thereby, specimens without neoadjuvant treatment showed lower performance with accuracy larger than 77% for the three classes, while specimens with neoadjuvant treatment revealed an accuracy of 86% for EAC and tumor stroma. Using the RGB channels only did not resulted in a satisfying accuracy (only 60% for EAC and tumor stroma), which demonstrated the superiority of our HSI analyses. Moreover, the statistical difference of the ROC-AUC-Score of the results from the several methods were calculated. The method using all patients with the HSI data versus all patients with synthesized RGB data showed for the class squamous epithelium a significant difference only.

**Table 1:** Classification results of the multi-layer perceptron method.

Classifier	EAC and tumor stroma [%]	Squamous epithelium [%]	Background [%]	Used dataset
Sensitivity	77 ± 24	54 ± 25	90 ± 45	All 95 patients
Specificity	84 ± 46	81 ± 22	97 ± 8	
Accuracy	78 ± 22	80 ± 20	97 ± 8	
F1-score	82 ± 20	53 ± 23	75 ± 41	
ROC AUC score	88 ± 46	71 ± 28	99 ± 49	
Sensitivity	80 ± 25	49 ± 33	88 ± 45	Without neodjuvant chemotherapy
Specificity	74 ± 48	81 ± 23	97 ± 4	
Accuracy	78 ± 22	77 ± 22	98 ± 4	
F1-score	83 ± 21	42 ± 30	76 ± 40	
ROC AUC score	90 ± 46	77 ± 39	99 ± 48	
Sensitivity	86 ± 22	65 ± 29	71 ± 33	With neodjuvant chemotherapy
Specificity	91 ± 26	87 ± 21	98 ± 4	
Accuracy	86 ± 21	85 ± 21	98 ± 4	
F1-score	89 ± 18	58 ± 37	70 ± 33	
ROC AUC score	94 ± 14	72 ± 27	100 ± 0	
Sensitivity	60 ± 31	32 ± 20	82 ± 43	With synthetic RGB
Specificity	76 ± 44	69 ± 23	88 ± 19	
Accuracy	60 ± 29	65 ± 24	88 ± 20	
F1-score	66 ± 28	30 ± 18	59 ± 35	
ROC AUC score	83 ± 44	47 ± 25	99 ± 48	

**Figure 5:**
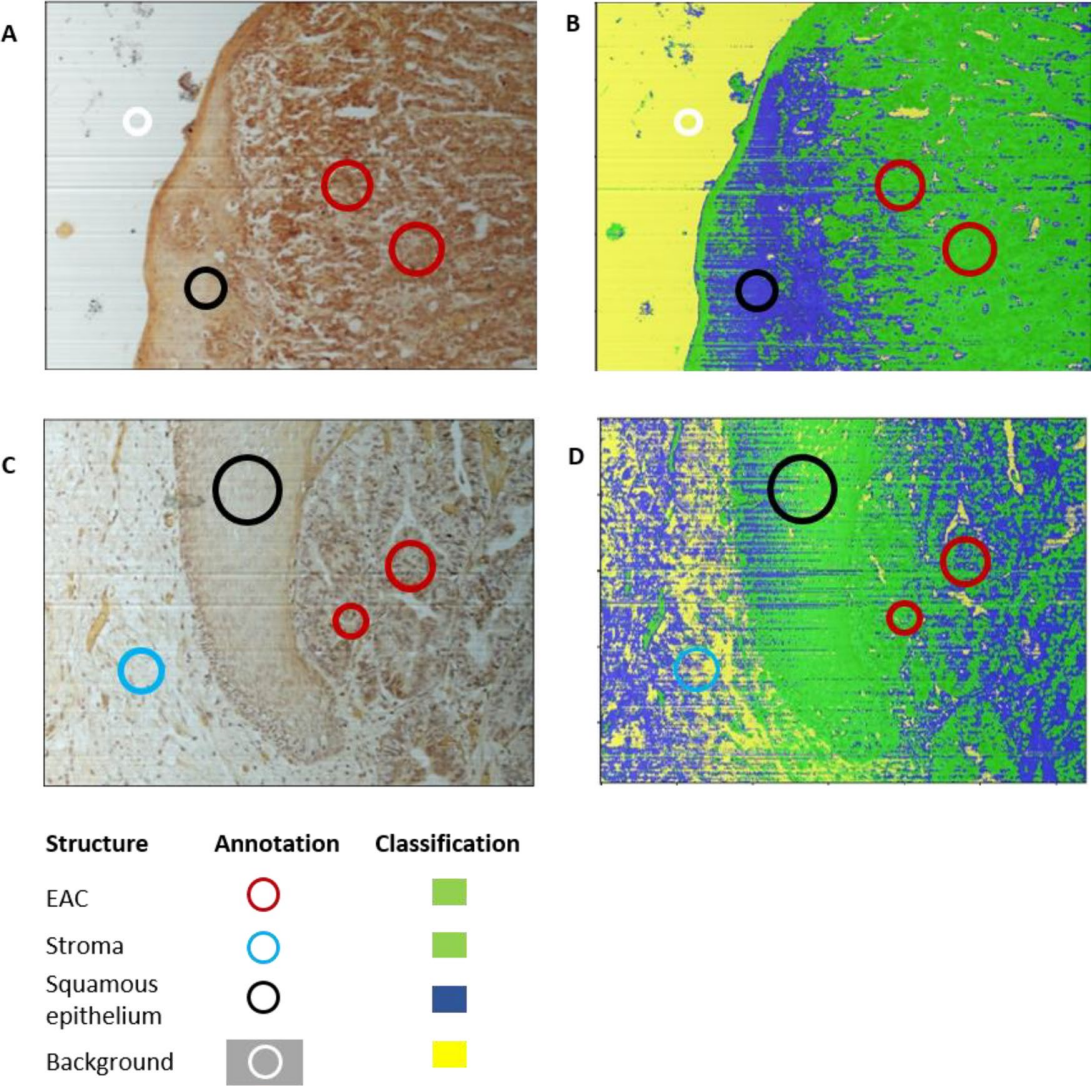
Visualization of the classification results. RGB image of an image (**A**) and the classification results visualized in (**B**). The RGB image (**C**) was classified and the classification results were visualized in, representing a visual misclassification, e.g. squamous epithelium was classified as tumor stroma (**D**).

Representative figures of the classification results are shown (Fig. 5). A correct classification was defined as weighted accuracy for all classes higher than 60%. A correct classification was obtained in 67 out of 95 specimens (71%). Figure 5A/B shows a classification example, in which the cells classified as EAC and stroma are depicted in green, squamous epithelium in blue, and background in yellow. In 22% of the specimens, the classification algorithm resulted in a less accurate classification (weighted accuracy for all classes lower than 60%) (Fig. 5C/D).

**Figure 6:**
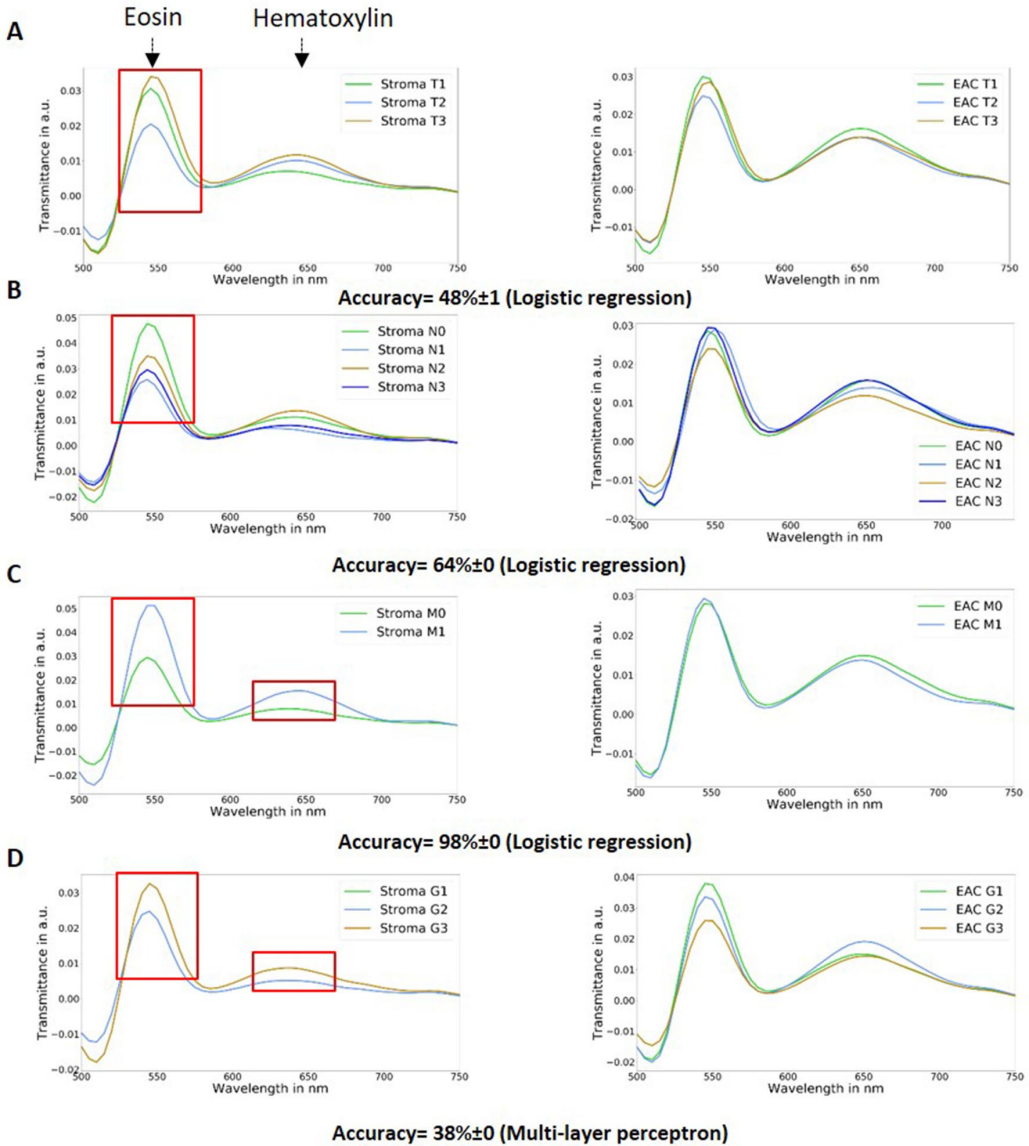
Mean transmittance of the TNM-classification (**A**–**C**) and grading (**D**) in stroma (first column) and EAC cells (second column). The achieved accuracy and the applied classification algorithm is shown under the spectra. The red rectangle shows the areas of high differences of the spectra. (**A**) The mean transmittance of stroma depicts high difference in the range from 525 nm to 575 nm in case of T-category only. (**B**) The mean transmittance of stroma reveals high variance in the range from 525 nm to 575 nm in case of the N-category. (**C**) The mean transmittance of stroma showed high variance in the range from 525 nm to 575 nm and in the range from 625 nm to 675 nm in case of the N-category. (**D**) The mean transmittance of stroma demonstrated high variance in the range from 525 nm to 575 nm and in the range from 625 nm to 675 nm in case of the N-category. The classification results for the gradings was less than 48% accuracy.

In this study, the patient cohort had a high variability in grading and TNM–categories. To analyze the influence on the spectra regarding these differences, several machine learning methods were tested and a tenfold-cross validation was used: Logistic Regression, Random Forrest, Multi-layer perceptron, and a Support Vector Machine with poly kernel to automatically discriminate between tumor categories and grading. Only 30% of the patients in each fold were used as test set to avoid overfitting. The classes EAC and tumor stroma were merged into one class (tumor tissue). The spectra of the tumor stroma and EAC cells as well as the accuracy score of the best classifier are described for TNM-categories and grading (G). Accuracy scores of 64% and 98% were achieved for the N- and M-categories. The logistic regression was the best classifier, whereby each classifier showed high performance values in case of M-category-classification. However, there were only 4 patients being M-positive versus 91 patients having no distant metastases. The T-classification and grading failed to show a high accuracy (accuracy less than 48%) (Fig. 6).

## Discussion

Digital pathology is an emerging field, which promises automated identification of tumor cells and deeper insights into tumor heterogeneity. While traditional microscopy relies to RGB images, HSI offers a technology using up to 100 spectral channels including wavelengths not visible for the human eye. Combined with HSI, AI algorithms will bring "Big Data" into microscopy.

In our current study, HSI was used to analyze specimens from esophageal adenocarcinoma patients, investigating tumor and squamous epithelium cells. Hyperspectral (HS) or multispectral (MS) imaging have been used for tumor cell identification in other gastrointestinal malignancies, e.g. gastric cancer and pancreatic cancer^12,13^. Thereby, slides were stained with HE and visualized by different HSI systems. However, the methods used for automatic tumor cell identification are highly diverse, and there has been no standard established so far. Also, the number of included patients differs. Hu et al. investigated 30 gastric cancer patients, which resulted in a sensitivity of 96%^13^. Ishikawa et al. included 12 patients only and used data of the spectral range from 420 to 750 nm to achieve a sensitivity of 91%^20^. In our study, data of 95 EAC patients were included to obtain an overall average classification accuracy of 78%, with specificity of 84% for the discrimination of tumor cells, and 81% for squamous epithelium cells.

In contrary to this study, Ishikawa et al. and Hu et al. used wavelengths from 350 nm to 1000 nm. In these studies more spectral values were obtained by using higher amounts of pixels in the images^13,20^. Possibly, better performance of the used MLP algorithms can be achieved by using a larger area of the HSI image for the labeling of several structures. Furthermore, in this study and in Hu et al., a realistic LOPOCV was done^13^. This work was based on a previous study, which had shown the best performance for tumor cell identification by a neuronal network with a MLP algorithm^21^. Additionally, SVM and LR models have been used. By evaluating the data in a grid seach cross-validation a MLP showed the highest sensitivity of 90%^21^. The sensitivity for the cohort investigated here was 77% for EAC and tumor stroma cells. Stronger learning algorithms will need more patient data, as the specificity and sensitivity of a classification is highly dependent on the intra- and inter-tumor diversity, as previously shown for glioblastomas^22^. Interesting will be the evaluation of patients’ prognosis and treatment response. Therefore, a prospective trial must be done, including biopsies obtained during patients’ staging procedure. Hu et al. showed that the spectral features of gastric cancer tissue have influence on the individual patient, and the spectral features of patients are varied. Maybe, this fact can explain that Ishikawa et al. achieved a high sensitivity of 92% even though 12 patients were included only^13,20^.

HSI camera systems are limited in conventional microscopy, as wavelengths over 750 nm are not able to pass the optics due to the utilized glass. However, in HE stained specimens the absorption is highly dependent on the tissue staining. In our cohort, the use of synthetic RGB images in the automatic classification has proved a lower accuracy than the results obtained with HSI data (Table 1). However, it should be mentioned that we have used a wavelength range from 500 to 1000 nm only with the result that the blue channel was generated by values between the ranges from 530 to 560 nm. In Halicek et al., a synthetic RGB image showed higher performance, but in Lu et al. and Qi et al., the spectral data reflected better results than synthesized as well as standard bright-field RGB data^18,23,24^.

The HSI camera detects the different HE staining capacity of cell structures and cell types. Cancer cells with their higher nucleus to plasma rate have a stronger staining than squamous epithelium, as these cells lost their nucleus during stratification and are less stained by eosin. Eosin has an absorption maximum at 525 nm and hematoxylin a peak at 630 nm. EAC cells and squamous epithelium cells, thus, showed a characteristic absorption pattern for hematoxylin and eosin. Therefore, the compositions of the achieved spectra are dependent on the used staining dyes and their intensity. Another staining method, other than HE, could be used to increase the information diversity. The usage of Masson Goldner staining or a periodic acid-shiff staining might improve the data quality. However, HE staining represents the most simple and reliable procedure to investigate specimens histopathologically.

The eosin and hematoxylin spectra play a crucial role to detect the different structures. Different staining conditions, e.g. staining time or temperature, can influence the color of the slides enormously^25^. To minimize these effects in our study, the staining conditions were standardized, and the slides were stained by one and the same technician. Albeit, some stained slides showed lower classification results than other slides. These misclassification results can be explained by coloring of the slides. In future studies, these facts can be more considered, e.g. by using methods which are described in^26^.

The used MLP algorithm is a feedforward network, which has been used in histopathology classification of brain cancer^27^ and in mitosis detection in breast cancer^28^. MLP is a complex algorithm, which provides fast (less than 10 s with a standard PC hardware) classification of the whole image size after training the model. The data set was unbalanced (e.g., EAC had 83% more data than squamous epithelium). To achieve high-quality and reproducible classification results, a balancing of the data set was necessary. Unfortunately, the numbers of possible spectra for training data for our model was thereby reduced (e.g., 18% of the patients had annotated squamous epithelium). In future studies, the annotation of the classes should be more balanced to achieve more spectral information of each patient and of each structure. Currently, spectral databases of pathological slides are rare. Therefore, the dataset cannot be extended. Nevertheless, further studies should evaluate, if transfer-learning methods are possible to improve classification outcomes. Moreover, the usage of autoencoder to increase the dataset can be studied in future. Some augmentation methods are already evaluated in the remote sensing area using hyperspectral data^29–31^.

Furthermore, our specimens reflected the whole range of pTNM-categories, which might have an influence on the spectra. The classification of the pTNM-categories and the grading was done and showed a high accuracy of up to 98% for the M-category, however, taking into account the low number of included M-positive-specimens. Furthermore, the pTNM-categorization might influence the HSI image by the several cell structures and their changed eosin and hematoxylin staining. As shown in Fig. 6, the spectra of EAC cells and tumor stroma differed with regard to the pTNM-category and grading. Whether HSI together with artificial intelligence is able to predict TNM-categories and grading, needs to be investigated in more detail in future studies. However, an influence of neoadjuvant treatment on the classification performance has been demonstrated. The classification test in patients without neoadjuvant treatment showed a higher (at least 13%) performance than the whole cohort studied and the patients with neoadjuvant treatment. Our results suggest that the differentiation of specimens with and without preoperative chemotherapy is relevant.

Our current data clearly show a robust tumor and squamous epithelium cell classification using a neuronal network with a MLP algorithm. AI algorithms will revolutionize standards in medical diagnostics. HSI is a promising and innovative technique, which allows an image acquisition beyond RGB images. The underlying data cubes contain a multiple data set as compared to those from conventional RGB images. Further studies with more spectral data, a balanced dataset, as well as developments of HSI combined with microscopy will be necessary, before an automated tumor cell identification algorithm can be used in daily clinical practice.

Classification with multispectral data and using SVM offers promising approaches^32^. For routine application, a simple but robust algorithm is needed to reduce time especially for examination of frozen sections during surgery, but the algorithm also needs to be efficient with an excellent specificity and sensitivity. In addition, a robust algorithm must cover the identification of precancerous lesions (Barrett’s metaplasia, low- and high-grade intraepithelial neoplasia) and carcinoma in situ in the future.

## Materials and methods

### Patients

Patients with histologically confirmed EAC were eligible for this study. The study was performed monocentrally, comprising a total of 95 patients between 2014 and 2017. After a standard oncologic *en bloc* esophagectomy specimens were processed. The study is in accordance with the declaration of Helsinki and the protocol was approved by the local ethic’s committee of the University of Leipzig (No. of the approval: 307–15). The ethic’s committee of the Landesaerztekammer Hesse adopted this approval without further requirement of informed consent for this study. The pTNM-categories were determined according to the 8th edition of the TNM classification of malignant tumours^33^.

### Study population

In total, 95 esophageal adenocarcinoma patients, who had undergone oncologic esophagectomy in the Sana Clinic Offenbach, were included in this study. The demographic and histopathological characterizations of all patients are shown in Table 2.

**Table 2:** Clinico-pathological patient’s characteristics.

Characteristics	Number of cases
Number	95
Median age; range (years)	63; 34–81
Gender	
Male	80
Female	15
T-category	
T1	45
T2	21
T3	29
T4	0
N-category	
N0	55
N +	40
M-category	
M0	91
M +	4
Grading	
G1	3
G2	13
G3	28
n.a.	51
Neoadjuvant treatment	
CTx	46
RCTx	3
n.a.	46

n.a. – not applicable; CTx – chemotherapy; RCTx—radiochemotherapy.

### Tumor specimens

After esophagectomy, tumor specimens (*n* = 95) from EAC patients were examined by experienced upper gastrointestinal pathologists (M.B. and S.B.), fixed in 4% formaldehyde, dehydrated and paraffin embedded (Sakura Tissue-Tek VIP 6, Staufen, Germany). For the HSI analyses, slices of 3 µm were conducted and stained with hematoxylin and eosin (HE) in a standardized manner on a glass slide (Superfrost Plus, Menzel-Gläser, Brunswig, Germany) and covered by cover slips (Menzel-Gläser, Brunswig, Germany). The histological slides were embedded in Mountex (Medite, Burgdorf, Germany). We assume that this substance does not have a specific peak in the wavelength area from 500 to 750 nm. All specimens were evaluated by at least one expertized pathologist (M.B. and S.B.), who confirmed tumor cell presence into the specimens used, were responsible for the TNM, R, G and pathological response rate classification, and confirmed the tumor cell annotation. In this study, there were no patients, which had a complete pathological response rate after chemotherapy.

### HSI recording

**Figure 7:**
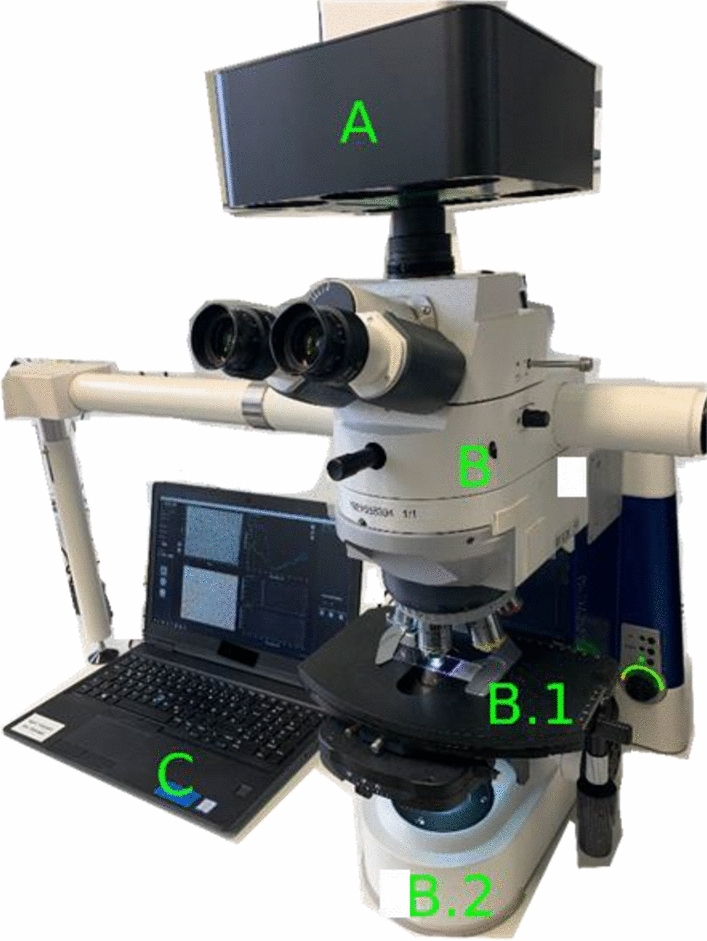
Experimental setup. The HSI camera (**A**) was fixed via a c-mount to a standard optical microscope (**B**). A halogen lamp (HAL100) was used (B.2) and stained slides were fixed to the specimen stage (B.1). A 20 × objective was used. HSI data were processed by a laptop computer (**C**).

The HE stained slices were recorded with a HSI camera system (Diaspective Vision GmbH, Am Salzhaff-Pepelow, Germany), which was mounted to a standard microscope (AxioVision, Carl Zeiss Microscopy GmbH, Jena, Germany). The HSI system recorded a hyperspectral data cube with the dimension: 640 × 480 (x-, y-axis) × 100 spectral channels over a wavelength range of 500 to 1000 nm, and a theoretical spatial resolution of 0.6 µm/pixel. Due to the glass lenses of the optical microscope, the used range for analyses was reduced to 500 to 750 nm. The microscope was equipped with a halogen lamp (100 W, HAL100, Carl Zeiss Microscopy GmbH, Jena, Germany) and the 20 × objective (Carl Zeiss Microscopy, Jena, Germany) was used (Fig. 7).

**Figure 8:**
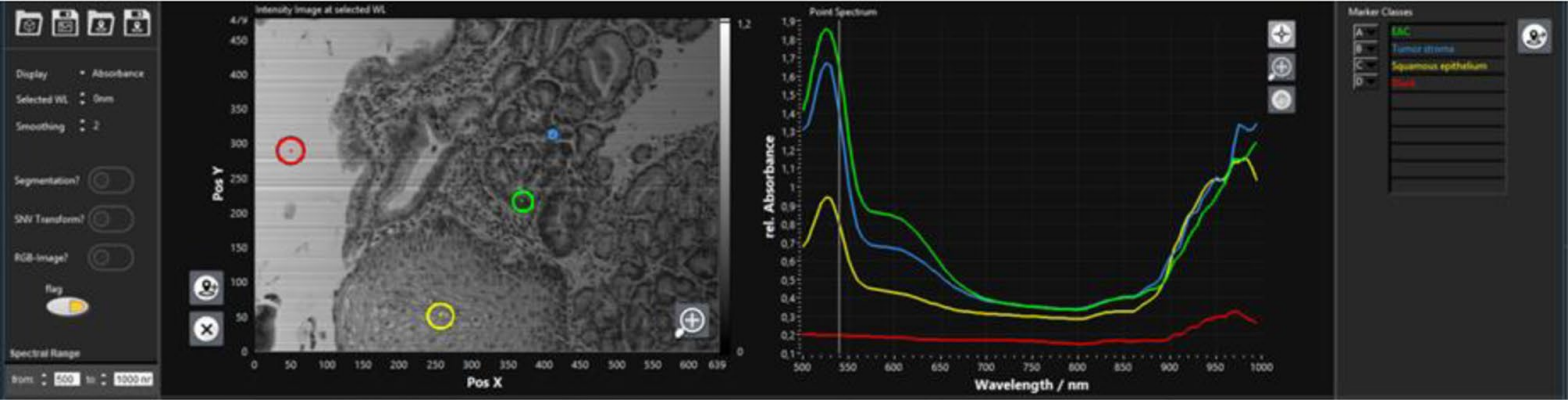
Annotation of EAC and squamous epithelium cells. Annotation was done to specify EAC cells (green), tumor stroma (blue), squamous epithelium (yellow), and blank/background (red). The left panel shows the RGB image provided by the TIVITA software to perform the annotations and the right depicts the corresponding averaged and smoothed spectra. Above wavelengths of 750 nm, the noise in the spectra was very high, which was caused by the lenses inside the microscope (e.g. glasses from lamps, objective).

The TIVITA Suite software was applied for recording the transmission spectra and performing the tissue annotation. Before the first measurement, a black and white balancing was done and the light source including the illumination intensity was kept constantly. Further details have been described by Kulcke et al.^34^. The images were stored as three-dimensional hypercubes. The RGB images reconstructed by the software were used for tissue annotation (Fig. 8). Thereby, at least 5 to 10 cells were annotated for all region of interests (ROI) for each category on the synthetic RGB image (Fig. 5 and 8). The annotations were performed by medical experts and reviewed by an experienced pathologist. Based on the position and radius of the ROIs, the corresponding spectra were extracted. Four classes were determined (squamous epithelium, EAC cells, tumor stroma cells, and blank or background).

### Feature extraction

**Figure 9:**
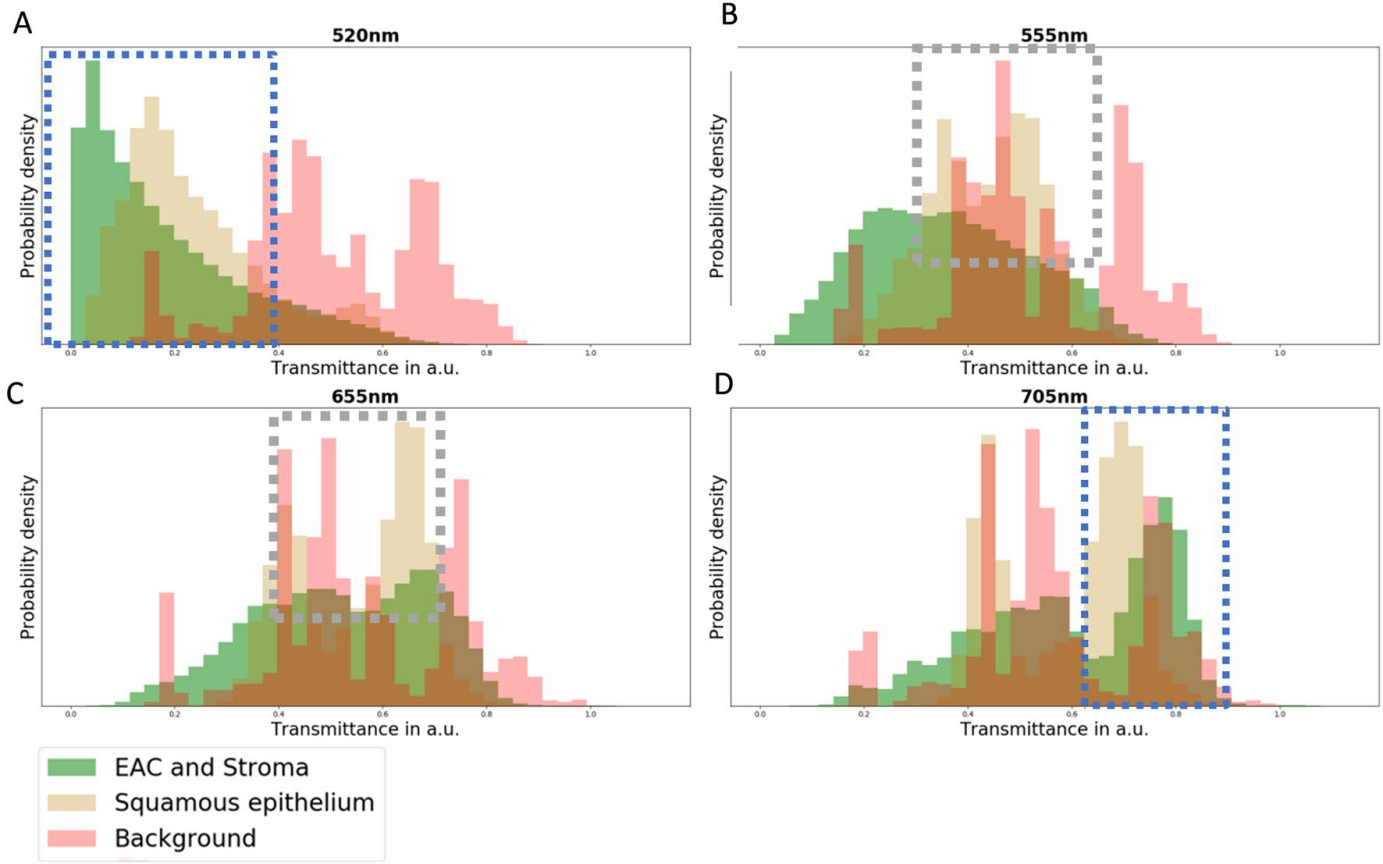
The histograms of the transmittance values of specific wavelengths are shown. For 520 nm (**A**) and 705 nm (**D**), the spectra of EAC, stroma, and squamous epithelium showed a high similarity in the probability densities (blue dotted rectangle). For the wavelengths, 655 nm (**C**) and 555 nm (**B**), the spectra of EAC and stroma against squamous epithelium showed differences in the probability density (grey dotted rectangle). Hence, the spectra values between 560 and 675 nm were used to classify these four structures. (a.u. aberrante units)

HE staining is the standard staining procedure in daily routine and tumor cell determination. The spectra of HE^12^, which were used to stain the specimens, have specific spectral features, with the highest absorbance at 524 nm for eosin, and at 630 nm for hematoxylin, respectively (Fig. 2). Analyzes of the histograms from 500 to 750 nm were done. The spectral area had to be ascertained in a range, where EAC, stroma cells as well as squamous epithelium had the highest difference in their transmittance values. We visually analyzed the histograms. Therefore, we considered the distribution of the classes and their probability densities. A big difference between the densities and less overlay of the classes was preferred, as a high-class difference was assumed. In conclusion, the wavelength range between 560 to 675 nm was used to include the information of the spectra of hematoxylin (Fig. 9). The spectral values below 560 nm and higher than 675 nm were excluded for further analyses, due to their high noise ratio, as well as the high similarity of the spectra from squamous epithelium cells, EAC, and tumor stroma cells (Fig. 2). In order to highlight the influence of the stains eosin and hematoxylin more accurately, the classification was performed, using eight spectral features:

$${{\varvec{E}}}_{1}=\boldsymbol{ }\frac{{{\varvec{S}}}_{{\varvec{k}}=560\boldsymbol{ }{\varvec{n}}{\varvec{m}}}}{{{\varvec{S}}}_{{\varvec{i}}=\boldsymbol{ }650\boldsymbol{ }{\varvec{n}}{\varvec{m}}}},\dots ,{{\varvec{E}}}_{6}=\boldsymbol{ }\frac{{{\varvec{S}}}_{{\varvec{k}}=585\boldsymbol{ }{\varvec{n}}{\varvec{m}}}}{{{\varvec{S}}}_{{\varvec{i}}=\boldsymbol{ }675\boldsymbol{ }{\varvec{n}}{\varvec{m}}}}$$

$${E}_{7}= \frac{1}{n}\sum_{k=1}^{n}{S}_{k}$$

$${E}_{8}= \frac{1}{n}\sum_{i=1}^{n}{S}_{i}$$

*E* is the calculated feature, *S*_*k*_ is the transmittance value of the *k*th band, and *S*_*i*_ is the transmittance value of the *i*th band. The variable *k* represents the bands between 560 and 585 nm, and variable *i* represents the bands between 650 and 675 nm with a resolution of 5 nm. Furthermore, SNV was used to normalize the spectra. A Gaussian filtering with a sigma of one were used to reduce the noise of the spectra.

Furthermore, synthesized RGB data were determined based on the HSI data to perform a comparison classification with the spectral data with the same image resolution. The three channels of the synthesized RGB image were calculated as the averaged transmittances on the following three wavelength ranges:

- 530 nm to 560 nm (as blue channel),
- 540 nm to 590 nm (as green channel), and
- 585 nm to 725 nm (as red channel).

Unfortunately, a blue channel was not recorded with the used HSI system. Therefore, we did not used the typical wavelengths for the blue and green channels. Thus, we calculated only a synthesized RGB image. The feature extraction was performed with the python library scikit-learn (version 0.23, https://scikit-learn.org). The mean spectra plots, the histograms were performed with python library matplotlib (version 3.4, https://matplotlib.org) and the PCA were performed with python library plotly (version 4.12, https://plotly.com/python).

### Image processing and data classification

**Figure 10:**
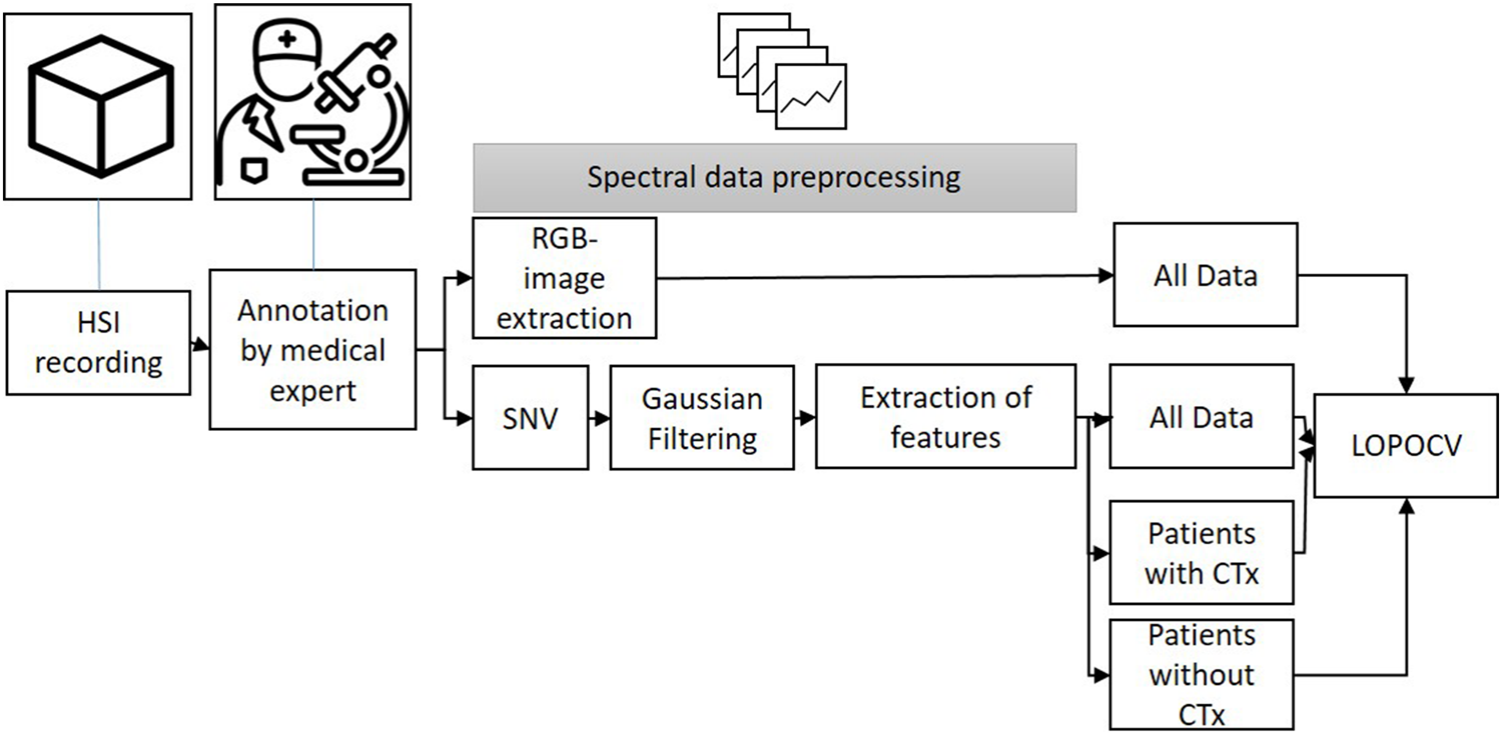
Schematic description of the data preprocessing (HSI- hyperspectral imaging, SNV- standard normal variate, RGB- Red Green Blue, CTx- neoadjuvant chemotherapy, LOPOCV- leave-one-patient-out cross-validation).

The pipeline of the preprocessing steps are shown in Fig. 10. The classification and image processing was performed with the python library scikit-learn (version 0.23, https://scikit-learn.org)^35^ and with python library matplotlib (version 3.4, https://matplotlib.org). To find the best classification model, the python library Scikit-Optimize (version 0.9.0, https://pypi.org/project/scikit-optimize) was used. In an extensive grid search using a Bayesian optimization, we determined the best-performing hyperparameters for each model (LR, SVM, and MLP).

For the classification of the HSI data, a multi-layer perceptron (MLP) was used. MLP are commonly used in HSI-classification tasks. For example, in Halicek et al., MLP was used to classify HSI data of specimens of thyroid and salivary tissues with tumor^24^. In our study, the MLP-input was performed with eight spectral features, extracted from the HSI data and three features from the synthesized RGB data, as defined in the previous section. The output were the tissue classes.

The network had two hidden layers with 32 and 16 nodes. The weights, which connect the nodes of the network, were calculated with a set of feature vectors based on an Adam solver. The Adam solver is a stochastic optimization method^36^. Each node of the network performs a hyperbolic tangent as activation function, which is appropriate for the spectrum data, whose values were in the range of -1 to 1. The calculations were done on a 64 GB RAM and 2.6 GHz processor machine with a balanced data set. The training took a few hours and the testing on the patient took a few seconds only.

A balanced data set was achieved by randomly choosing the same number of spectra of EAC, tumor stroma, and background as the number of spectra of squamous epithelium.

The performance of classification was evaluated with a LOPOCV for each of the patients (e.g. 95 times) and the following three classes: EAC and tumor stroma (class one), squamous epithelium (class two), and background (class three). To measure the performance of the neuronal network, five standard statistical measures were used. The calculation was done pixel-wisely. The MLP produces a score for each class, where a higher score indicates a greater likelihood of the tissue class being present at the spatial coordinate. The class with the highest predictive score of the pixel was selected. This process was repeated for each pixel of interest. The specificity (the quotient of the sum of the true negatives and the sum of condition negatives), the sensitivity (the quotient of the sum of the true positives and the sum of condition positives) and the accuracy (the sum of the true positive and the true negatives divided by the total population) were calculated. These measurements were used for the multi-class and binary classification problems. The Receiver Operator Curve Area-Under-Curve (ROC-AUC) was calculated. ROC-AUC provides the probability that a random positives example (e.g. EAC and stroma) was scored higher with a model as compared to a random negative example (e.g. background and squamous epithelium). ROC-AUC summarized the recognition performance with a single number that was not tied to a specific decision threshold. Furthermore, the F1-Score, also called Sorensen-Dice-coefficient (DICE), was used. It measured the harmonic mean of precision and recall.

To compute statistically significant differences, a two-tailed paired t-test (p = 0.05) similar to previous works in HSI classification analysis using Excel (Microsoft Office 365, Microsoft, Redmond, WA, USA), was used^37^.

## Data Availability

The datasets and codes generated during the current study are available from the corresponding author on reasonable request.
